# Effect of Acute Cadmium Exposure and Short-Term Depuration on Oxidative Stress and Immune Responses in *Meretrix meretrix* Gills

**DOI:** 10.3390/toxics14010047

**Published:** 2025-12-31

**Authors:** Yu Zheng, Yijiao Zheng, Xuantong Qian, Yinuo Wu, Alan Kueichieh Chang, Xueping Ying

**Affiliations:** 1 College of Life and Environmental Sciences, Wenzhou University, Wenzhou 325035, China; 13906867874@163.com (Y.Z.); 19016500109@163.com (Y.Z.); 19032560919@163.com (X.Q.); yinuo_wu0507@163.com (Y.W.); 2 National and Local Joint Engineering Research Center of Ecological Treatment Technology for Urban Water Pollution, Wenzhou University, Wenzhou 325035, China

**Keywords:** cadmium, *Meretrix meretrix*, gill, oxidative stress, immune responses

## Abstract

Cadmium (Cd) is a typical pollutant with strong toxicity even at low concentrations. In the marine environment, Cd is a problem of magnitude and ecological significance due to its high toxicity and accumulation in living organisms. The clam *Meretrix meretrix* is a useful bioindicator species for evaluating heavy-metal stress. This study investigated the extent of recovery from Cd^2+^-induced oxidative and immune impairments in *M. meretrix* gills achieved by short-term depuration. Clams were exposed to 3 mg/L Cd^2+^ for six days or three days followed by three days of depuration, and the Cd contents, morphological structure, osmoregulation, oxidative stress, and immune responses in the gills were evaluated. The results showed that gill Cd contents increased with exposure, reaching 9.857 ± 0.074 mg·kg^−1^ on day 3 but decreased slightly to 8.294 ± 0.056 mg·kg^−1^ after depuration, while reaching 18.665 ± 0.040 mg·kg^−1^ on day 6 after continuous exposure. Histological lesions, including lamellar fusion, hemolymphatic sinus dilation, and ciliary degeneration, partially recovered after depuration. Reactive oxygen species (ROS) and malondialdehyde (MDA) levels decreased significantly, while DNA-protein crosslinking rate (DPC) and protein carbonyl (PCO) showed minor reductions. Total antioxidant capacity (T-AOC) and the activities of Ca^2+^/Mg^2+^-ATPase (CMA), cytochrome c oxidase (COX), succinate dehydrogenase (SDH), and lactate dehydrogenase (LDH) increased by over 10% during depuration, though these changes were not statistically significant. Lysozyme (LZM) activity and MT transcript levels increased progressively with Cd exposure, indicating their suitability as biomarkers of Cd stress. Acid and alkaline phosphatase (ACP, AKP) activities and *Hsp70* and *Nrf2* mRNA transcripts exhibited inverted U-shaped response consistent with hormetic response. ACP and AKP activity levels rose by more than 20% after depuration, suggesting partial restoration of immune capacity. Overall, Cd exposure induced oxidative damage, metabolic disruption, and immune suppression in *M. meretrix* gills, yet short-term depuration allowed partial recovery. These findings enhance understanding of Cd toxicity and reversibility in marine bivalves and reinforce the usage of biochemical and molecular markers for monitoring Cd contamination and assessing depuration efficiency in aquaculture environments.

## 1. Introduction

In recent decades, the marine ecosystems have faced serious heavy metal pollution because of increased industrial and other human-made activities along the coasts [1,2]. Cadmium (Cd), which has little involvement in physiological activities, can cause severe toxicity even at low concentrations [1,3,4]. In 2015, Cd was designated by the Poison Control Board of the United States as the sixth toxic non-essential metal among dangerous substances endangering human health [5]. Over the past few decades, Cd inputs in many industrialized and rapidly urbanizing coastal and semi-enclosed marine areas have remained elevated and, in some cases, increased [6,7,8]. In unpolluted water, the level of dissolved Cd generally ranges from 10 to 500 ng L^−1^, but levels that exceed 10 µg L^−1^ have been recorded in the water near industrialized areas in China [1,6,9]. The sources of Cd inputs are mainly anthropogenic and include mining and smelting, fertilizer and pigment industries, shipyards, wastewater discharge, and river-borne runoff. These localized inputs have created Cd “hotspots” in sediments and in high-accumulating seafood such as bivalves, cephalopods, and seaweeds, where ecological risk is measurable and where frequent consumption can approach or exceed health-based intake limits [10,11,12,13]. However, this pattern is not global, as Cd inputs to the marine environment have stabilized or even declined in several regions (especially the NorthEast Atlantic) under environmental regulations [14,15,16]. Consequently, current human-health concerns arise mainly from regional seafood chains linked to coastal contamination.

In marine environments, Cd readily accumulates in filter-feeding bivalves, which are widely used as bioindicators for monitoring environmental quality [17,18]. Cadmium exposure disrupts cellular redox balance, damages macromolecules, interferes with ion transport, and impairs immune function, leading to complex toxicological effects at multiple biological levels [19,20]. Thus, bivalves have become a valuable tool to study the toxicity of cadmium in the attempt to elucidate the path and mechanism of such toxicity. Beyond its implication for human health, investigating Cd toxicity in bivalves helps to clarify the biological pathways of metal accumulation and defense, supporting better monitoring, pollution control, and seafood safety practices.

We have previously reported that short-term depuration of the Cd-exposed clam, *Meretrix meretrix*, can alleviate Cd-induced oxidative damage in the ovary of the clam through partial metal elimination and activation of antioxidant defense [21]. However, reproductive tissues are not the initial site of Cd entry, and their response reflects downstream accumulation rather than direct uptake and first-line defense. By contrast, gills are in direct contact with seawater and represent the primary interface for Cd absorption, osmoregulation, and respiratory exchange, making them a key target for both acute toxicity and detoxification processes. Despite this, the temporal sequence and molecular mechanisms of Cd-induced injury and partial recovery in gills remain insufficiently understood.

Furthermore, most previous studies have focused on general oxidative stress markers (e.g., ROS, MDA, T-AOC), providing a phenomenological view of toxicity. There is still limited understanding of how redox regulatory pathways, mitochondrial function, ion transport, and immune enzymes interact to govern reversible versus irreversible damage during exposure and depuration. This gap hinders the development of reliable biomarker systems and depuration protocols tailored to different tissues and time windows.

This current study aimed to elucidate the biochemical, molecular, and histological mechanisms underlying Cd-induced toxicity and partial reversal through short-term depuration in the gills of *M. meretrix*. Clam *M. meretrix* is a common and economically farmed shellfish in China, and thus Cd toxicity in these animals will subsequently affect human health through food consumption. In this study, we integrated a comprehensive suite of oxidative stress indicators, mitochondrial and ion transport enzymes, immunome hydrolases, and gene expression profiles (*MT*, *Hsp70*, *Nrf2*) to identify reversible versus irreversible damage endpoints during depuration and to reveal the regulatory pathways coordinating oxidative, metabolic, and immune responses in a tissue that serves as the first barrier and detoxification interface. By focusing on gill-specific temporal dynamics and molecular regulation, this work complements previous studies on reproductive tissues and provides new insights into the spatial and mechanistic complexity of Cd toxicity in bivalves. These insights have direct implications for biomarker development and depuration strategies in marine pollution monitoring and shellfish aquaculture.

## 2. Materials and Methods

### 2.1. Clam Collection and Treatments

*Meretrix meretrix* individuals (mean shell length: 53.24 ± 0.74 mm; shell width: 26.63 ± 0.37 mm; shell height: 44.21 ± 0.61 mm; and body weight: 41.65 ± 1.78 g) were purchased from the Wengyang aquafarm in Wenzhou, Zhejiang, China. All experimental procedures were approved by Wenzhou University’s Animal Ethical and Welfare Committee (No. WZU-2025-147). The clams were randomly assigned to three groups: one control group and two Cd^2+^-treated groups. Each group consisted of 180 clams, and these were kept in three separate aquariums (67 × 46 × 36.5 cm^3^, 60 clams per aquarium). For Cd^2+^ treatment, the clams were placed in aquariums filled with 15‰ artificial seawater containing 3 mg/L Cd^2+^ (equivalent to 1/5 of the 96 h Median Lethal Concentration (9LC_50_) [22]). The two Cd^2+^-treated groups were designated as T-1 and T-2. The T-1 group was exposed to Cd^2+^ for six days, whereas the T-2 group was exposed to Cd^2+^ for three days, followed by depuration in Cd^2+^ free seawater for another three days. The control group was maintained in 15‰ artificial seawater for six days. For each group, the water in the tank was completely replaced with fresh water daily without Cd^2+^ or with 3 mg·L^−1^ Cd^2+^ as required. No food was provided to the clams during the treatment period, and all other conditions were kept the same as during the acclimation phase (22 ± 1 °C, pH 8.0). The aquarium water was replaced daily with fresh 15‰ artificial seawater, either with or without Cd^2+^ as required, and water samples and gill tissues were collected every 24 h to determine their Cd^2+^ concentrations.

### 2.2. Detection of Cd Content in Water and Gill of Meretrix meretrix

The Cd^2+^ level in the water was determined according to Chen et al. [17]. The Cd^2+^ content in the gill was also determined as described for the determination of Cd^2+^ content in sediment in the same study [17] with slight modification. Instead of sediment, 2 g of gill tissue was mixed with 10 mL of solution containing 9 mL HNO_3_ (analytical grade) and 1 mL HClO_4_ (analytical grade) inside a 50 mL porcelain crucible with a lid. The crucible was kept at room temperature for overnight to allow the digestion to proceed. After that, the lid of the crucible was removed and the sample was heated at 150 °C for 1 h, followed by further heating at 180 °C until the clear and transparent solution was reduced to 2–3 mL. After that, the sample was processed as previously described [17]. The concentrations of Cd^2+^ in both water and gill tissue samples were measured using an atomic absorption spectrophotometer (Shimadzu Corporation AA-6300, Kyoto, Japan). The LOD and LOQ values for the measurement were 0.0018 mg/L and 0.0060 mg/L, respectively.

### 2.3. Histological Assays of Gill Specimens

Fresh *M. meretrix* gills collected every 24 h were fixed in formaldehyde, then embedded in paraffin, sectioned, stained, and sealed following the standard protocol described by Bai et al. [19]. The sections were examined and photographed using an Olympus BX51 microscope (Olympus, Tokyo, Japan).

### 2.4. Oxidative Stress and Immune Biochemical Indicator Assays

To measure oxidative stress and immune biochemical parameters, five clams were randomly selected from each tank every 24 h. Their gills were extracted, homogenized, and centrifuged according to the method of Zhou et al. [21]. Oxidative damage indices (MDA, DPC, PCO, ROS, T-AOC), ATP-metabolizing enzymes (NKA, CMA), mitochondrial marker enzymes (COX, SDH, LDH), and immunohydrolases (ACP, AKP, and LSZ) were determined using commercial assay kits (Nanjing Jiancheng Biological Engineering Institute, Nanjing, China) according to the manufacturer’s instructions.

### 2.5. MT, HSP70, and Nrf2 mRNA Expression Levels Analysis

Three clams were randomly selected from each tank every 24 h, and their gills were collected for total RNA extraction. Total RNA extraction, RNA quality, reverse transcription of RNA into cDNA, and quantitative PCR (qPCR) were performed as previously described for *M. meretrix* ovaries [21], and the sequences of the *MT* primers were taken from that study whereas the sequences of the *Hsp70* and *Nrf2* primers were designed based on the results obtained from the transcriptome sequencing of *M. meretrix* gills. The sequences of these primers are as follows: MT-F: 5′-CGAGGACTGTTCATCAACCACTG-3′; MT-R: 5′-GCAAACAACTTTACACCCTGGAC-3′; HSP70-F: 5′-CCACCAAGCAGACACAGAC-3′; HSP70-R: 5′-CGTTCAGGATACCGTTAGCAT-3′; Nrf2-F: 5′-GGTGTAGGGAAGGAAGTGTT-3′; and Nrf2-R: 5′-CACTCTCCAGTTTCTCCATCT-3′. Quantitative expression analysis was performed according to the method of Choi et al. [23], and the relative expression levels were calculated using the 2^−ΔΔCt^ method.

### 2.6. Data and Statistical Analyses

Statistical analyses were performed with the SPSS (Version 23.0, Chicago, IL, USA) and OriginPro (Version 9.2, Northampton, MA, USA). A one-way analysis of variance (ANOVA) was conducted with sampling day as the fixed factor to identify significant differences within each treatment group, followed by Tukey’s post hoc test. All data were presented as the mean ± standard error (SE, n = 3).

## 3. Results

### 3.1. Changes in Cd^2+^ Levels in the Water and M. meretrix Gills During Cd^2+^ Exposure

The Cd^2+^ concentrations in the water of the control group remained consistently low (0.001–0.003 mg/L) throughout the six days of treatment with no significant differences (*p* > 0.05) among sampling days (Table 1). In the T-1 group, water Cd^2+^ concentrations dropped to about half the initial concentration (3 mg/L Cd^2+^) after four days and then increased on days 5–6, suggesting a greater absorption of Cd^2+^ by the clams on days 1–4. In the T-2 group, water Cd^2+^ concentration showed the same profile as in the T-1 group for days 1–3, reminiscent of Cd^2+^ uptake. However, the significantly higher water Cd^2+^ concentrations on days 4–6 compared with the control group indicate the release of Cd^2+^ from the clams following the cessation of exposure, consistent with the onset of depuration. A similar profile was also evident in the case of Cd^2+^ contents in the gills. In the control group, gill Cd^2+^ contents remained low and showed no notable variation over time (0.208–0.215 mg/kg) (Table 1). In both T-1 and T-2 groups, gill Cd^2+^ contents increased during the first three days, reaching almost 10 mg/kg. However, from day 4 to day 6, gill Cd^2+^ contents in the T-1 group continued to increase, whereas those in the T-2 group decreased. The lower gill Cd^2+^ contents in the T-2 group relative to T-1 group during the later treatment days indicate the loss of accumulated Cd^2+^ from the gill tissues, further reflecting depuration activity following the cessation of waterborne Cd^2+^ exposure.

Together, the higher water Cd^2+^ concentrations and decreasing gill Cd^2+^ levels in the T-2 group during days 4–6 indicate active Cd^2+^ release from the clams, demonstrating that short-term depuration reduced Cd^2+^ retention in both the water and gill tissues compared with the T-1 group, confirming the capacity of *M. meretrix* for detoxification when Cd exposure is lifted.

### 3.2. Changes in Gill Structure of M. meretrix During Different Days of Cd^2+^-Exposed

**Figure 1:**
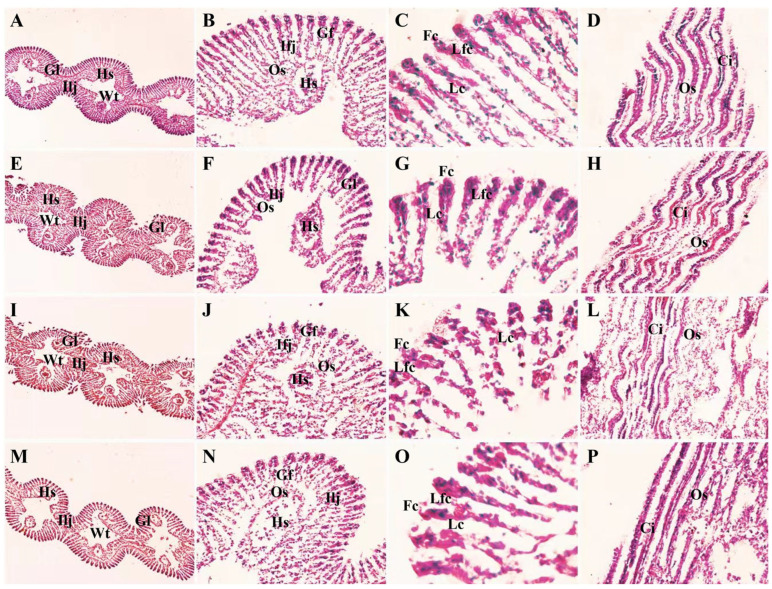
Changes in gill tissue structure of *Meretrix meretrix* under different days of Cd^2+^ exposure. Note: (**A**–**D**) control; (**E**–**H**) the 3rd day of the T-1 group; (**I**–**L**) the 6th day of the T-1 group; (**M**–**P**) the 6th day of the T-2 group. (**A**,**E**,**I**,**M**) are cross sections (10×); (**B**,**F**,**J**,**N**) are cross sections (40×); (**C**,**G**,**K**,**O**) are cross sections (100×); (**D**,**H**,**L**,**P**) are scuttled (40×). Gill lamella (Gl), Hemolymph sinuses (Hs), septal valve (Ilj) and Water tube (Wt); Gill filaments (Gf), septal filaments (Ifj), and branchial foramina (Os), anterior cilia (Fc), anterior cilia (Lfc), lateral cilia (Lc), cilia (Ci).

The gill filaments of non-exposed *M. meretrix* individuals were thin, closely arranged, and well defined (Figure 1A–D), with numerous cilia attached to the surface of the filaments (Figure 1C). In contrast, in the Cd^2+^-exposed clams, the lamellar structure of the gill tissue became deformed and loosely connected, and the number of blood lymphocytes in the hemolymph sinuses increased with prolonged Cd^2+^ exposure (Figure 1E–L). The gill filaments exhibited deformation, scattered arrangement, and fusion or fracture of adjacent lamellae, accompanied by severe ciliary detachment, and the extent of damage intensified by day 6 (Figure 1I–L). The gill filaments of the T-2 group on day 6 appeared clearer and more regularly arranged, with fewer blood cells present (Figure 1M–P), and the degree of structural damage was less than that observed in the T-1 group (Figure 1I–P). The results suggest that the severity of gill damage was dependent on exposure duration (T-1: irreversible structural collapse), and termination of Cd^2+^ exposure allowed partial epithelial reorganization (T-2: mitigated pathology), although complete functional recovery likely requires a longer depuration period.

### 3.3. Effects of Cd^2+^ on the Levels of MDA, PCO, and DPC in M. meretrix Gill

**Figure 2:**
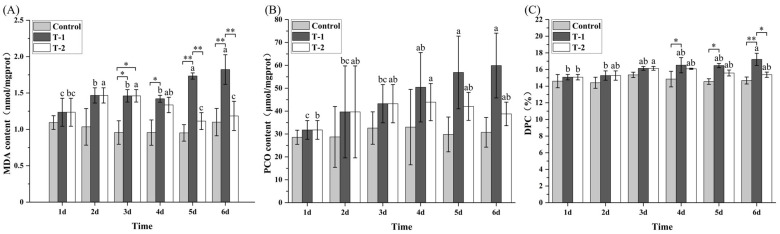
Effects of Cd^2+^ on the oxidative markers in *M. meretrix* gills. (**A**) MDA; (**B**) PCO; (**C**) DPC. Data are the means ± standard errors (n = 3). Different letters above the pillars indicate significant (*p* < 0.05) differences among different days for the same group (control group, T-1 group, or T-2 group) as revealed by Tukey’s post hoc multiple comparison tests. “*” and “**” indicate statistical significance among groups at the *p* < 0.05 and *p* < 0.01 levels, respectively.

In the control group, gill MDA, PCO, and DPC concentrations showed no significant differences (*p* > 0.05) throughout days 1–6 (Figure 2). In contrast, in both T-1 and T-2 groups, gill MDA concentrations progressively increased with Cd^2+^ exposure. In the T-1 group, MDA reached its highest concentration on day 6, which was significantly higher than that on days 1–4 (*p* < 0.05). In the T-2 group, MDA concentrations on days 5–6 were significantly lower than those on days 2–4 and were not significantly different from the concentration on day 1. Furthermore, on days 5–6, the T-1 exhibited significantly higher (*p* < 0.05) MDA concentrations than both the control group and T-2 groups (Figure 2A). For PCO, there was no significant difference between day 1 and either day 2 or 3 in the T-1 group, although the PCO concentrations on days 5 and 6 were significantly higher (*p* < 0.05) than those in the control group. In the T-2 group, the PCO concentration on day 4 was significantly higher than on day 1, whereas PCO concentrations on days 5–6 were lower than those in the T-1 group, but with no statistically significant differences (Figure 2B). For DPC, the concentration in the T-1 group on day 6 was significantly higher compared with days 1–2. The T-2 group showed a slight decline in DPC concentrations during days 5–6, but no statistically significant differences were detected compared with days 1–3. Moreover, on day 6, the T-1 group exhibited significantly higher DPC concentrations than both the control group and T-2 groups (Figure 2C). These results demonstrate that Cd^2+^ exposure induced exposure-duration-dependent accumulation of oxidative damage, whereas cessation of exposure partially reversed membrane and protein damage but failed to completely repair DNA crosslinks, highlighting persistent genomic instability as a critical residual risk.

### 3.4. Effects of Cd^2+^ on the Levels of ROS and T-AOC in M. meretrix Gill

**Figure 3:**
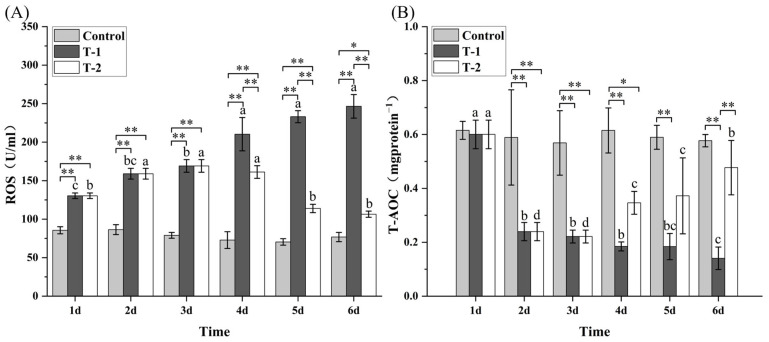
Effect of Cd^2+^ on *M. meretrix* gill ROS (**A**) and T-AOC (**B**) concentrations. Note: Data are the means ± standard errors (n = 3). Different letters above the pillars indicate significant (*p* < 0.05) differences among the different groups (control group, T-1 group, and T-2 group). “*” and “**” indicate significant differences at the *p* < 0.05 and *p* < 0.01 level, respectively.

Gill ROS and T-AOC concentrations in the control group showed no statistically significant differences throughout days 1–6 (*p* > 0.05; Figure 3). In the T-1 group, gill ROS concentration increased progressively with prolonged Cd^2+^ exposure, reaching its peak on day 6, and was significantly higher (*p* < 0.05) than those on days 1–3. In contrast, the T-2 group exhibited significantly lower (*p*< 0.01) ROS concentrations during days 5–6 compared with days 2–4. During days 4–6, ROS concentrations in the T-1 group were significantly elevated (*p* < 0.01) relative to both the control and T-2 groups (Figure 3A).

In the T-1 group, the gill T-AOC decreased progressively with prolonged Cd^2+^ exposure, reaching its minimum on day 6, and values during this period were significantly lower (*p* < 0.05) than those on days 1–4 (Figure 3B). Conversely, the T-2 group displayed an upward trend in T-AOC concentrations during days 4–6, with the value on day 6 being significantly higher (*p* < 0.05) than those on days 2–5 yet significantly lower than that on day 1. Notably, throughout days 4–6, T-AOC concentrations in the T-2 group remained significantly higher (*p* < 0.05) than in the T-1 group but significantly lower (*p* < 0.01) than in the control group (Figure 3B). These results indicate that Cd^2+^ exposure caused a dose/time-dependent collapse of the antioxidant system (T-1 group), whereas cessation of exposure led to partial redox recovery (T-2 group), although residual oxidative impairment persisted.

### 3.5. Effects of Cd^2+^ on the Activities of NKA and CMA in M. meretrix Gill

**Figure 4:**
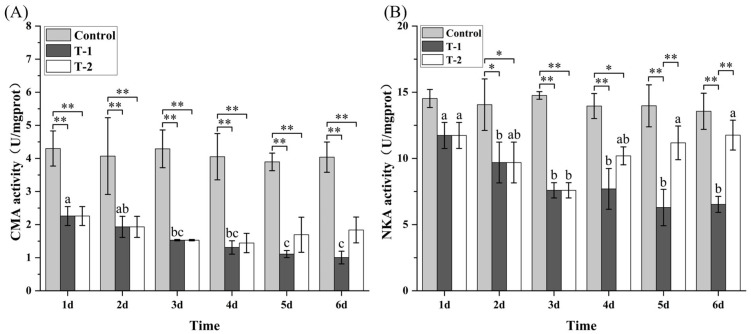
Effect of Cd^2+^ on the activities of CMA (**A**) and NKA (**B**). Note: Data are the means ± standard errors (n = 3). Different letters above the pillars indicate significant (*p* < 0.05) differences among the different Cd^2+^-treated groups (control group, T-1 group, and T-2 group) in the gills. “*” and “**” indicate significant differences at the *p* < 0.05 and *p* < 0.01 level, respectively.

The control group maintained stable gill Ca^2+^/Mg^2+^-ATPase (CMA) and Na^+^/K^+^-ATPase (NKA) activities across the six treatment days, and both enzyme activities were significantly higher than those of the T-1 and T-2 groups (Figure 4). In the T-1 group, CMA activity showed a declining trend, reaching its minimum on day 6, which was significantly lower (*p* < 0.05) than on days 1 and 2 (Figure 4A). In the T-2 group, CMA activity displayed a biphasic response—an initial decrease followed by partial recovery—although no statistically significant differences were detected across the six-day period. The persistent reduction in CMA activity in the T-1 group suggests irreversible proteostatic disruption, whereas the slight partial recovery in the T-2 group indicates some degree of adaptive capacity.

In the T-1 group, NKA activity showed a gradual decline with prolonged Cd^2+^ exposure, although no statistically significant differences were observed on days 2–6 (Figure 4B). The T-2 group exhibited a fluctuating pattern—an initial decrease followed by a later increase—and on day 6, the NKA activity was similar to that on day 6 but significantly higher (*p* < 0.05) than that on day 3. During days 5–6, NKA activity levels in the T-2 group were significantly higher (*p* < 0.05) than those in the T-1 group but remained lower than those in the control group (Figure 4B). The biphasic NKA response in the T-2 group suggests initial ionoregulatory disruption followed by compensatory upregulation, whereas the sustained decline observed in the T-1 group indicates progressive toxicity.

### 3.6. Effects of Cd^2+^ on COX, SDH, and LDH Activities in M. meretrix Gill

**Figure 5:**
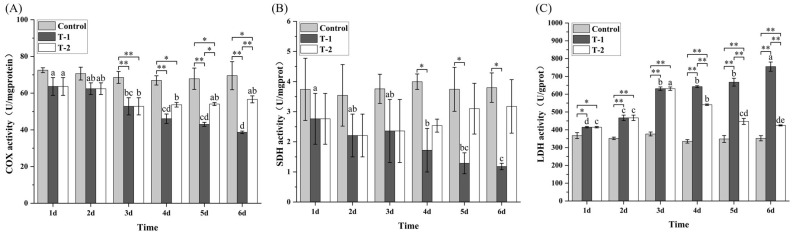
Effects of Cd^2+^ on COX (**A**), SDH (**B**), and LDH (**C**) activities in *M. meretrix* gills. Note: Data are the means ± standard errors (n = 3). Different letters above the pillars indicate significant (*p* < 0.05) differences among the different Cd^2+^-treated groups (control group, T-1 group, and T-2 group) in the gills. “*” and “**” indicate significant differences at the *p* < 0.05 and *p* < 0.01 level, respectively.

Overall, COX and SDH activities in the gills of the control group remained stable throughout the six treatment days and were higher than those in the two Cd^2+^-treated groups, though significant differences were detected only on some of the days (Figure 5A,B). In contrast, COX and SDH activities in the T-1 group showed progressive declines with increased exposure duration, reaching levels that were higher than those in the T-2 group on days 4–6. Both enzyme activities reached their minimum on day 6 and were significantly lower (*p* < 0.05) than those on days 1–3. In the T-2 groups, COX and SDH activities also declined initially through day 3, after which the activities on days 4–6 either stabilized or increased slightly compared with day 3, indicating no further deterioration. As for LDH activity, it remained constant in the control group and was significantly lower than those in the T-1 and T-2 groups (Figure 5C).

Across all three enzymes, the control group showed consistently stable activities, whereas the T-1 group displayed progressive inhibition of COX and SDH and continuous elevation of LDH. In contrast, the T-2 group showed stabilization or recovery in enzyme activities after day 3, with values on days 4–6 returning toward or remaining closer to the control levels. These patterns collectively indicate that depuration mitigated the enzymatic disturbances induced by Cd exposure, supporting the protective effect of the T-2 condition.

### 3.7. Effects of Cd^2+^ on the Activities of ACP, AKP, and LSZ in M. meretrix Gill

**Figure 6:**
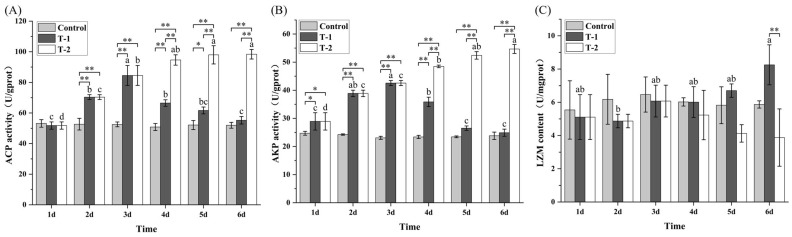
Effect of Cd^2+^ on the activities of hydrolases in *M. meretrix* gills. (**A**) ACP; (**B**) AKP; (**C**) LZM. Data are the means ± standard errors (n = 3). Different letters above the pillars indicate significant (*p* < 0.05) differences among the different Cd^2+^-treated groups (control group, T-1 group, and T-2 group) in the gills. “*” and “**” indicate significant differences at the *p* < 0.05 and *p* < 0.01 level, respectively.

No significant (*p* > 0.05) temporal changes in ACP, AKP, and LZM activities were detected in the control group throughout the 6-day treatment period (Figure 6). However, in the T-1 group, ACP and AKP activities showed an initial increase followed by a subsequent decrease, and by day 6, both dropped to levels not significantly different from those on day 1 (Figure 6A,B). In contrast, the T-2 group exhibited progressive elevation in both ACP and AKP activities over the treatment period, reaching maximal levels on day 6 that were significantly higher (*p* < 0.05) than those on day 3. During days 4–6, ACP and AKP activities in the T-2 group were significantly higher (*p* < 0.01) than those in both the T-1 and control groups. The biphasic phosphatase response in the T-1 group suggests initial lysosomal stress followed by cellular exhaustion, whereas the sustained elevation in the T-2 group indicates adaptive membrane remodeling.

Lysozyme (LZM) activity in the T-1 group peaked on day 6 and was significantly higher (*p* < 0.05) than the levels on days 1–5 (Figure 6C). The T-2 group exhibited a biphasic response in LZM activity, characterized by an initial increase followed by a decrease, with the minimal value on day 6 being significantly lower (*p* < 0.05) than those on days 1–4. On day 6, LZM activity in the T-1 group was significantly higher (*p* < 0.01) than in the T-2 group. The sustained elevation of LZM activity in the T-1 group indicates a chronic inflammatory response, whereas the decline in the T-2 group suggests that, even after removal of exogenous Cd^2+^ (day 4), intracellularly accumulated Cd^2+^ continued to impair lysosomal function, resulting in delayed LZM recovery.

### 3.8. Effect of Cd^2+^ on the Level of MT, HSP70 and Nrf2 mRNA in M. meretrix Gill

**Figure 7:**
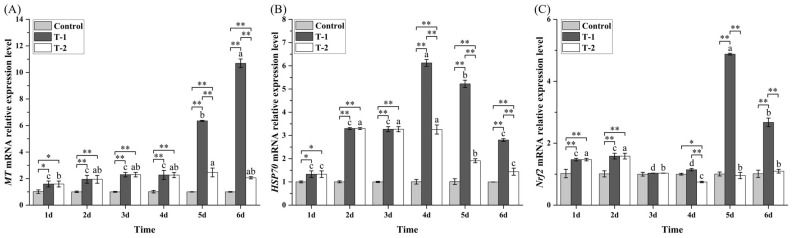
Effects of Cd^2+^ on the transcript levels of oxidative stress responsive gene. (**A**) *MT* mRNA; (**B**) *HSP70*; (**C**) *Nrf2.* Data are the means ± standard errors (n = 3). Different letters above the pillars indicate significant (*p* < 0.05) differences among the different Cd^2+^-treated groups (control group, T-1 group, and T-2 group) in the gills. “*” and “**” indicate significant differences at the *p* < 0.05 and *p* < 0.01 level, respectively.

The mRNA levels of *MT*, *HSP*, and *Nrf2* in the control group showed no statistically significant changes throughout the 6-day treatment period (Figure 7). In the T-1 group, the *MT* mRNA level increased modestly during days 1–4 and then surged sharply, peaking on day 6 at a level that was significantly higher (*p* < 0.05) than those on days 1–5 and markedly higher than the levels in both the control and T-2 groups on days 5–6 (*p* < 0.01) (Figure 7A). In the T-2 group, *MT* mRNA remained relatively stable at low-to-moderate levels across days 1–6, generally above the control but far below the T-1 values on days 5–6 (*p* < 0.01).

*Hsp70* mRNA in the T-1 group increased to a pronounced peak on day 4 and then declined on days 5–6, although the level on day 6 remained higher than that on day 1, though well below the day- 4 maximum (Figure 7B). In the T-2 group, Hsp70 mRNA was elevated on days 1–4 compared with the control (*p* < 0.05) and then decreased on days 5–6. Between groups, the T-1 and T-2 groups exhibited similar Hsp70 levels on days 1–3 but differed significantly on days 4–6 (*p* < 0.01), with the T-2 group showing at least 50% less *Hsp70* mRNA than the T-1 group, though *Hsp7* mRNA levels in both groups remained higher than those of the control group across all days.

In the T-1 group, *Nrf2* mRNA levels on days 3 and 4 were significantly lower than those on days 1 and 2 (*p* < 0.05) but rose significantly to a maximum on day 5 and decreased on day 6, which nevertheless remained significantly higher than days 1–4 (Figure 7C). In the T-2 group, *Nrf2* mRNA showed modest fluctuations, with relatively higher levels on days 1–2, followed by a drop on day 4 and a partial rebound on day 6. Between groups, T-1 showed markedly higher *Nrf2* mRNA levels than both the control and T-2 groups on days 5–6 (*p* < 0.01), with intergroup differences being comparatively small around day 3. On day 4, however, the T-1 group also exceeded the T-2 and control groups.

Taken together, these results reveal a phased transcriptional response to Cd^2+^ stress, whereby *Hsp70* responds early, *Nrf2* peaks slightly later (day 5 in T-1), consistent with redox-regulatory activation, and *MT* shows the strongest late-phase induction (day 5 in T-1), consistent with sustained, metal-binding detoxification.

## 4. Discussion

Cadmium (Cd^2+^) is a non-essential and highly toxic metal that readily bioaccumulates in aquatic organisms, posing risks to marine ecosystems and human consumers. In this study, *M. meretrix* was found to exhibit rapid Cd accumulation in the gills, confirming this organ as the primary site of uptake and early toxicity. The progressive increase in Cd^2+^ concentration with exposure time and its partial elimination during depuration demonstrate the clam’s capacity for metal sequestration and gradual detoxication. This trend is consistent with reports on *Crassostrea virginica* and *Mytilus galloprovincialis*, where Cd accumulation reached a plateau after prolonged exposure due to physiological saturation or epithelial injury [24,25,26]. The substantial reduction in Cd in the depuration group (T-2) indicates active efflux, possibly mediated by ATP-binding cassette transporters such as ABCC1 [27,28,29]. However, the elimination was incomplete, suggesting intracellular retention of Cd-metallothionein (MT) complexes within lysosomes [30,31,32], which explains the persistence of Cd residues after short depuration.

Histological deformation, lamellar fusion, and ciliary detachment observed in the Cd^2+^-exposed clams (Figure 1E–O) confirmed that Cd^2+^ directly damages gill architecture, consistent with earlier findings in *M. meretrix* [21], *Procambarus clarkii* [33] and *Anodonta woodiana* [34]. These lesions compromise respiration and osmoregulation and likely reduce subsequent metal uptake. The partial structural restoration following depuration demonstrates limited epithelial repair, although full functional recovery may require a longer duration. Comparable reversibility has been documented in *Gammarus fossarum* and *Grassostrea gigas* [4,35].

Prolonged exposure of *M. meretrix* to Cd induced oxidative stress characterized by elevated ROS, MDA, and PCO, DPC concentrations in the gill tissue (Figure 2 and Figure 3A). These heightened levels of oxidative stress markers confirm that Cd disrupts redox equilibrium by depleting antioxidants and impairing mitochondrial electron transport [19,36,37]. The progressive increase in MDA and ROS reflects cumulative oxidative injury, whereas the recovery of total antioxidant capacity (T-AOC) (Figure 3B) during depuration indicates reactivation of endogenous defense pathways. Similar redox recovery after Cd removal has been reported in *Anodonta woodiana* and *Cyprinus carpio* [38,39,40]. Nevertheless, persistent DPC elevation in the gill tissue (Figure 2C), even after depuration, implies irreversible nucleic acid damage and incomplete cellular repair.

Cadmium exposure significantly suppressed Na^+^/K^+^-ATPase (NKA) and Ca^2+^/Mg^2+^-ATPase (CMA) activities (Figure 4), indicating ion-regulatory failure and reduced ATP production. Comparable inhibition has been reported in clams and crabs, where Cd binds to the catalytic sites of ATPase and disrupts transmembrane ion gradients [41,42,43]. The partial recovery of NKA activity during depuration suggests that upregulation might have been compensated once Cd stress subsides. Cadmium also decreased cytochrome c oxidase (COX) and succinate dehydrogenase (SDH) activities while elevating lactate dehydrogenase (LDH) activity (Figure 5), consistent with previous reports [36,44], signifying a shift from aerobic to anaerobic metabolism. The underlying basis likely includes Cd^2+^ interference with the heme a_3_/Cu(B) binuclear center of COX [45]. Together, these findings highlight mitochondrial impairment and energetic depletion as key mechanisms of Cd^2+^-induced dysfunction, with recovery dependent on reactivation of oxidative phosphorylation.

The biphasic trends in acid phosphatase (ACP), alkaline phosphatase (AKP), and lysozyme (LZM) activities reflect an early activation of cellular defense systems followed by metabolic exhaustion under prolonged Cd exposure [46]. The early rise in ACP suggests enhanced lysosomal digestion and autophagy, while the rise in AKP likely supports membrane-associated detoxification and ion-transport processes [47]. Although no bacterial challenge was conducted, the increase in LZM activity probably represents a generalized stress-induced immune response aimed at clearing damaged cells and debris [48], analogous to defenders responding to the aftermath of toxic assault rather than to invading pathogens. The subsequent decline in all three enzyme activities during sustained Cd exposure may result from ATP depletion and structural impairment of lysosomal and membrane systems, leading to reduced hydrolytic and immune capacity. During depuration, the partial restoration of ACP, AKP, and LZM activities (Figure 6) indicates metabolic recovery and reestablishment of cellular homeostasis. Similar transient immune modulation under heavy-metal stress has been reported in other bivalves [49], suggesting that these enzymes serve as reliable biomarkers of both stress intensity and recovery status.

At the molecular level, Cd exposure elicited a sequential stress-response cascade, which included early *Hsp70* induction, mid-term *Nrf2* activation, and late *MT* upregulation. The transient *Hsp70* elevation indicates early chaperone-mediated protection against misfolded proteins [50,51]. Strong *MT* induction highlights its role in Cd sequestration and ROS neutralization [52,53]. Increased *Nrf2* expression confirms activation of the antioxidant signaling axis that coordinates phase II detoxification [28,54]. The downregulation of *Nrf2* after depuration reflects negative feedback via Keap 1 and reduced ROS stimuli [55]. Collectively, these patterns reveal a tightly regulated network linking redox control, metal detoxification, and immune modulation in clam gills.

By integrating histological, biochemical, and molecular endpoints, we have elucidated the temporal dynamics of Cd toxicity and recovery in *M. meretrix*. The findings show that oxidative stress and mitochondrial impairment underpin gill injury, while antioxidant and detoxification pathways confer partial resilience. Cd-induced damage appears at least partially reversible within the 3-day exposure period examined; however, longer or repeated exposures could exceed this compensatory capacity. Future work extending exposure and depuration durations will be necessary to determine the precise threshold beyond which recovery becomes limited, ideally under conditions that minimize confounding effects such as starvation stress. Furthermore, the results reinforce the applicability of biomarkers such as MDA, *MT*, and *Hsp70* as sensitive indicators of heavy-metal stress and recovery status in marine bivalves, supporting their continued application in environmental monitoring and aquaculture safety assessment.

Although this study reveals key biochemical and molecular events associated with Cd toxicity and short-term recovery, it is limited by the brief experimental duration. The three-day exposure and depuration design was deliberately chosen to induce observable physiological responses without causing mortality, as longer trials in unfed clams tend to introduce confounding stress from starvation-related stress even when water quality is maintained by daily renewal. Moreover, evidence from previous studies on *A. woodia* [39] indicates that extending depuration beyond several days does not necessarily enhance Cd elimination, even though prolonged exposure continues to promote accumulation. This suggests that Cd removal in bivalves is inherently slow and constrained by intracellular sequestration mechanisms such as Cd-MT complex formation. Consequently, future research should therefore focus not merely on extending depuration but also on elucidating the cellular and molecular pathways that limit Cd efflux and MT turnover.

## 5. Conclusions

This study provides an integrated view of cadmium-induced injury and recovery in the gills of *Meretrix meretrix*, a tissue that functions as both the primary interface for metal uptake and the first line of detoxification. By combining oxidative stress biomarkers, mitochondrial and ion-transport enzyme activities, immune hydrolases, and gene expression profiling, we distinguished between reversible and persistent effects of short-term Cd exposure. The results reveal a coordinated regulatory network linking redox homeostasis, energy metabolism, and immune response, underscoring the gill’s central role in systemic adaptation to metal stress. When considered alongside our previous work on reproductive tissues, these findings extend understanding of the tissue-specific complexity of Cd toxicity in bivalves and offer a mechanistic basis for interpreting biomarker response across organs. Ultimately, this integrative framework enhances our capacity to evaluate depuration efficiency and to refine biomarker-based strategies for environmental monitoring and sustainable shellfish aquaculture.

## Figures and Tables

**Table 1:** Content (mg/L) and concentration (mg/kg) of Cd in *Meretrix meretrix* gills during different treatment days.

Days		1	2	3	4	5	6
Water	Control	0.002 ± 0.001 ^b^	0.001 ± 0.003 ^b^	0.003 ± 0.003 ^b^	0.002 ± 0.003 ^c^	0.003 ± 0.004 ^c^	0.002 ± 0.004 ^c^
	T-1	1.428 ± 0.058 ^Ca^	1.458 ± 0.035 ^Ca^	1.442 ± 0.037 ^Ca^	1.460 ± 0.033 ^Ca^	1.676 ± 0.007 ^Ba^	1.865 ± 0.040 ^Aa^
	T-2	1.401 ± 0.052 ^Aa^	1.463 ± 0.031 ^Aa^	1.419 ± 0.035 ^Aa^	0.174 ± 0.001 ^Bb^	0.168 ± 0.009 ^Bb^	0.134 ± 0.007 ^Cb^
Gills	Control	0.208 ± 0.002 ^b^	0.211 ± 0.001 ^b^	0.211 ± 0.005 ^b^	0.215 ± 0.003 ^c^	0.209 ± 0.004 ^c^	0.213 ± 0.004 ^c^
	T-1	6.915 ± 1.021 ^Da^	8.622 ± 0.047 ^Ca^	9.842 ± 0.037 ^Ca^	13.460 ± 0.033 ^Ba^	17.376 ± 0.027 ^Aa^	18.665 ± 0.040 ^Aa^
	T-2	6.928 ± 1.007 ^Ca^	8.458 ± 0.035 ^Ba^	9.857 ± 0.074 ^Aa^	9.122 ± 0.086 ^Ab^	8.696 ± 0.084 ^Bb^	8.294 ± 0.056 ^Bb^

Note: Data are the means ± standard errors (n = 3). Different uppercase letters indicate significant differences among different days under the same group, whereas different lowercase letters indicate significant differences among different Cd^2+^ treatments on the same day at *p* < 0.05. T-1 group: treated with Cd^2+^ for six days; T-2 group: treated with Cd^2+^ for three days followed by depuration in Cd^2+^ free seawater for another three days.

## Data Availability

All authors guarantee that all data and materials support our published claims and the Data are available by contacting X.Y. (xpying2008@wzu.edu.cn).

## Abbreviations

ACP	Acid phosphatase
AKP	Alkaline phosphatase
Cd	Cadmium
CMA	Ca^2+^/Mg^2+^-ATPase
COX	Cytochrome c oxidase
DPC	DNA-protein crosslinking rate
Hps 70	Heat shock proteins 70
LDH	Lactate dehydrogenase
LZM	Lysozyme
MDA	Malondialdehyde
MT	Metallothionein
NKA	Na^+^/K^+^ ATPase
Nrf2	Nuclear factor E2-related factor 2
PCO	Protein carbonyl
ROS	Reactive oxygen species
SDH	Succinate dehydrogenase
T-AOC	Total antioxidant capacity
